# HIF-1alpha Deficiency Attenuates the Cardiomyogenesis of Mouse Embryonic Stem Cells

**DOI:** 10.1371/journal.pone.0158358

**Published:** 2016-06-29

**Authors:** Jana Kudová, Jiřina Procházková, Ondřej Vašiček, Tomáš Perečko, Miroslava Sedláčková, Martin Pešl, Jiří Pacherník, Lukáš Kubala

**Affiliations:** 1 Institute of Biophysics, Academy of Sciences of the Czech Republic, Brno, Czech Republic; 2 Institute of Experimental Biology, Faculty of Science, Masaryk University, Brno, Czech Republic; 3 International Clinical Research Center – Centre of Biomolecular and Cellular Engineering, St. Anne's University Hospital, Brno, Czech Republic; 4 Department of Histology and Embryology, Faculty of Medicine, Masaryk University, Brno, Czech Republic; 5 Department of Biology, Faculty of Medicine, Masaryk University, Brno, Czech Republic; 6 International Clinical Research Center, St. Anne's University Hospital, Brno, Czech Republic; University of Kansas Medical Center, UNITED STATES

## Abstract

Cardiac cell formation, cardiomyogenesis, is critically dependent on oxygen availability. It is known that hypoxia, a reduced oxygen level, modulates the *in vitro* differentiation of pluripotent cells into cardiomyocytes via hypoxia inducible factor-1alpha (HIF-1α)-dependent mechanisms. However, the direct impact of HIF-1α deficiency on the formation and maturation of cardiac-like cells derived from mouse embryonic stem cells (mESC) *in vitro* remains to be elucidated. In the present study, we demonstrated that HIF-1α deficiency significantly altered the quality and quantity of mESC-derived cardiomyocytes. It was accompanied with lower mRNA and protein levels of cardiac cell specific markers (myosin heavy chains 6 and 7) and with a decreasing percentage of myosin heavy chain α and β, and cardiac troponin T-positive cells. As to structural aspects of the differentiated cardiomyocytes, the localization of contractile proteins (cardiac troponin T, myosin heavy chain α and β) and the organization of myofibrils were also different. Simultaneously, HIF-1α deficiency was associated with a lower percentage of beating embryoid bodies. Interestingly, an observed alteration in the *in vitro* differentiation scheme of HIF-1α deficient cells was accompanied with significantly lower expression of the endodermal marker (hepatic nuclear factor 4 alpha). These findings thus suggest that HIF-1α deficiency attenuates spontaneous cardiomyogenesis through the negative regulation of endoderm development in mESC differentiating *in vitro*.

## Introduction

The differentiation and maturation of cardiac progenitors into beating cardiomyocytes is a complex process controlled by close interactions between specific signaling pathways, transcription factors, and the cellular microenvironment. Deregulation of these processes leads to the generation of pathological states in the heart [1]. Studies demonstrate the essential role of oxygen availability, one of the critical factors regulating cardiomyogenesis during embryonal and postnatal development [2, 3]. Interestingly, physiological embryonic heart development occurs under conditions of low oxygen availability, or hypoxia. However, the exposure of the adult heart to hypoxia is involved in a variety of pathological conditions such as heart failure and myocardial infarction [4].

Hypoxia triggers the transcription of a large number of genes involved in many cellular processes including glycolysis, angiogenesis, apoptosis and cell proliferation with the purpose of minimizing deleterious effects caused by oxygen insufficiency at the cellular, tissue, and systemic levels [5]. The activation of these genes is generally mediated by hypoxia inducible factors (HIFs), transcription factors which belong to the basic helix-loop-helix-Per-Arnt-Sim family [6]. HIFs are heterodimeric protein complexes consisting of an oxygen-independent β subunit and an oxygen-dependent α subunit with tightly regulated protein stability and activity. Three HIF-α factors–HIF-1α, HIF-2α and HIF-3α have been recognized [7]. HIF-1α and HIF-2α are closely related in their protein structures and both recognize common HIF responsive elements [8]. However, they differ in their tissue expression profiles. While HIF-1α expression is present ubiquitously under hypoxic conditions in any tissue, HIF-2α expression is identified prominently in vascular endothelial cells during embryonic development and in kidney, liver, heart, brain, and lung [9].

The complete deficiency of HIF-1α (HIF-1α^-/-^) in mice results in an embryonic lethality of around E10.5 and is manifested by severe abnormalities including neural fold defects, angiogenesis dysfunction, and cardiac malformations [10]. In contrast, HIF-2α knockout mice (HIF-2α^-/-^) die at around the seventh week after birth due to metabolic defects such as mitochondrial dysfunction and abnormalities in liver, heart, and skeletal muscles [11]. On the other hand, *in vitro* studies revealed positive effects of ectopically expressed HIF-1α [12] or HIF-2α [13] in mouse embryonic stem cells (mESC) undergoing cardiomyogenesis. Interestingly, mice with the conditional knockout of HIF-1α in ventricular cardiomyocytes were shown to be viable but to display defects in cardiac function, vascularity, energy availability, and calcium handling [14]. However, a subsequent study by *Krishnan et al*. suggested the essential role of HIF-1α in ventricular cardiomyocyte development via the deregulation of genes associated with cardiac morphogenesis and contractility, which resulted in functional irreversible changes that eventually led to embryonic lethality of between E11.5 and E12.0 [2].

Since these *in vivo* data confirm the deleterious impact of HIF-1α deficiency on heart tissue development and function, it is therefore of particular interest to describe more precisely the role this key hypoxia sensor plays in the processes of cellular fate determination during stem cell differentiation and during cardiomyogenesis, in particular.

For this reason, we employed an *in vitro* model of spontaneously differentiated mESC with wild type (HIF-1α^+/+^) and knocked out (HIF-1α^-/-^) HIF-1α gene expressions. A detailed study of the influence of HIF-1α deficiency on cardiomyogenesis may help to increase general understanding of the molecular mechanisms of various cardiovascular diseases and to improve cardiac regeneration therapy.

## Materials and Methods

### mESC cultivation

The mESC lines HIF-1α^+/+^ (cell line R1) and HIF-1α^-/-^ were kindly provided by Peter F. Carmeliet of the Vesalius Research Center, VIB, University of Leuven, Belgium. The cells were thoroughly characterized according to their cytokinetic and phenotypic profiles as was shown [15]. It was shown that both parental and HIF-1α^-/-^ cells proliferate at similar rates. The cells were cultivated on gelatin-coated dishes in Dulbecco’s modified Eagle’s medium (DMEM; HyClone; Logan, UT, USA) supplemented with 15% fetal bovine serum (Gibco; Carlsbad, CA, USA), 100 IU/ml penicillin and 0.1 mg/ml streptomycin (Sigma; St. Louis, MO, USA), 1x non-essential amino acid (Gibco; Carlsbad, CA, USA), 0.05 mM β-mercaptoethanol (Fluka; Buchs, Switzerland), and 1 000 U/ml of leukemia inhibitory factor (Chemicon; Temecula, CA, USA). The cells were maintained at 37°C in humidified air supplemented with 5% CO_2_.

### mESC differentiation

**Fig 1:**
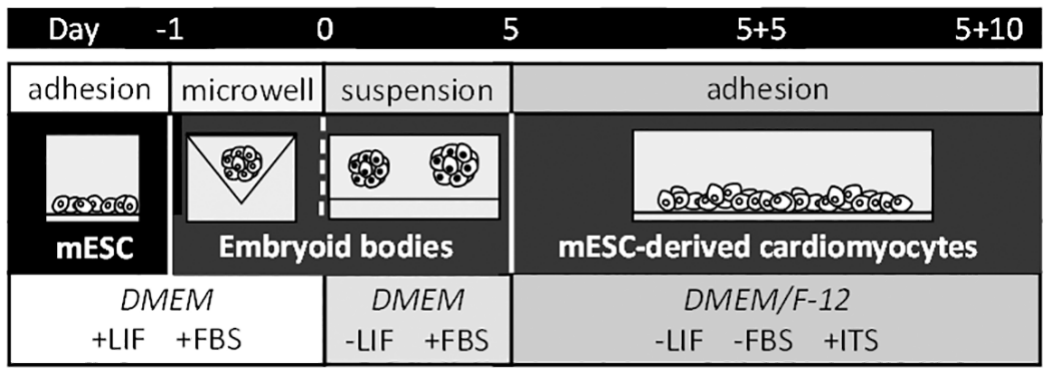
Schematic illustration of the protocol used for the *in vitro* differentiation of mESC mESC- mouse embryonic stem cells; DMEM- Dulbecco’s modified Eagle’s medium; LIF- leukemia inhibitory factor; FBS- fetal bovine serum; ITS- insulin-transferrin-selenium.

A suspension of mESC (2.5x106 cells/ml) was directly seeded on the top of microwells (1.5% agarose; VWR) to form embryoid bodies (EBs) of uniform size (for more details, see [16]). After 24 hours of incubation (day 0), the EBs were gently transferred onto an agar-coated dish and cultivated in medium without leukemia inhibitory factor. On day 5 (5d), the EBs were seeded on gelatin-coated dishes into DMEM/F-12 (1:1) medium (HyClone; Logan, UT, USA) supplemented with insulin-transferrin-selenium (Gibco; Carlsbad, CA, USA) and antibiotics (specification above) and cultivated for a further 5 (5+5d), 10 (5+10d) or 15 (5+15d) days (Fig 1). These time points represent various stages of cardiomyocyte development: cardiac progenitors (up to 5 days), early cardiomyocyte-like cells (up to 10 days), and beating cardiomyocyte-like cells (between 15 and 20 days). The stabilization of HIF-1α factor in early phases of the differentiation and the complete absence of the HIF-1α protein in HIF-1α^-/-^ was confirmed (S1 Fig).

### Gene expression analysis

Total RNA was extracted using the UltraClean Tissue & Cells RNA Isolation Kit (MO BIO Laboratories; Carlsbad, CA, USA). 1 μg of total RNA was reversely transcribed into first strand cDNA using the Transcriptor First Strand cDNA Synthesis Kit according to the manufacturer's protocol (Roche; Basel, Switzerland). Real-time quantitative PCR (RT-qPCR) reactions were performed in a LightCycler480 instrument using LightCycler480^®^ Probes Master solutions (Roche; Basel, Switzerland) and the following program: an initial denaturation step at 95°C for 10 min, followed by 45 cycles (95°C for 10 s, 60°C for 30 s, and 72°C for 1 s), and a final cooling step at 40°C for 1 min. Data were normalized to ribosomal protein L13A (Rpl13a) mRNA levels and presented as 2^-ΔΔcq^ [17]. The sequences of primers and numbers of Universal Probe Library (UPL) probes are listed in Table 1.

**Table 1:** Sequence of primers used in quantitative RT-PCR.

Gene of interest	Forward primer 5′→ 3′	Reverse primer 5′→ 3′	UPL probe no.
Rpl13a	CATGAGGTCGGGTGGAAGTA	GCCTGTTTCCGTAACCTCAA	# 25
Actn2	CCGGATTCTGGCTTCTGAT	GAGGCAGCTCTCGACGAA	# 53
Nkx2.5	GACGTAGCCTGGTGTCTCG	GTGTGGAATCCGTCGAAAGT	# 53
Myh6	CGCATCAAGGAGCTCACC	CCTGCAGCCGCATTAAGT	# 6
Myh7	CGCATCAAGGAGCTCACC	CTGCAGCCGCAGTAGGTT	# 6
Myl2	CCCAGATCCAGGAGTTCAAG	CTGCAGCCGCAGTAGGTT	# 95
Myl7	CCCATCAACTTCACCGTCTT	AACATGCGGAAGGCACTC	# 7
Hnf4α	CAGCAATGGACAGATGTGTGA	TGGTGATGGCTGTGGAGTC	# 27
Otx2	GGTATGGACTTGCTGCATCC	CGAGCTGTGCCCTAGTAAATG	# 55
Brachyury	ACTGGTCTAGCCTCGGAGTG	TTGCTCACAGACCAGAGACTG	# 27
Oct4	GTTGGAGAAGGTGGAACCAA	CTCCTTCTGCAGGGCTTTC	# 95
Nanog	GCCTCCAGCAGATGCAAG	GGTTTTGAAACCAGGTCTTAACC	# 25
αSMA	GACACCACCCACCCAGAG	ACATAGCTGGAGCAGCGTCT	# 20
Glut1	GGACCCTGCACCTCATTG	GCCACGATGCTCAGATAGG	# 20
Tie2	TGCCCAGATATTGGTGTCCT	CAGTGTGGTGTTCACGTATGTCT	# 11
VE-cad	GCAGTGGAGAGAGCCTTCTG	CTGTGAGCCTCTGCATCCTT	# 25
Flk1	CAGTGGTACTGGCAGCTAGAAG	ACAAGCATACGGGCTTGTTT	# 68
Vegf	CA GGCTGCTGTAACGATGAA	GCTTTGGTGAGGTTTGATCC	# 9
Gata1	GCCTGTCAGCCATCTTATGC	GTGGGGAAGGAGGTTGTAGG	# 3
PU.1	ATGGAGAAGCTGATGGCTTG	GGAACTGGTACAGGCGAATC	# 27

### Protein expression analysis

Total protein lysates were prepared from undifferentiated mESC or from mESC differentiated for particular time intervals (0d, 1d, 2d, 5d, 5+5d, 5+10d) using a lysis buffer (50 mM Tris-HCl, 100 mM NaCl, 10% glycerol, 1% SDS, 1mM EDTA, proteases and phosphatases inhibitors (Roche; Basel, Switzerland) at pH 7.4). Protein concentrations were determined using BCA Protein Assay (Pierce Biotechnology; Rockford, IL, USA) according to the manufacturer’s instructions. Approximately 15 μg (HIF-1α) or 20 μg (MHCα/β detected by MF20 antibody, cardiac troponin T (cTnT), α-actinin, octamer-binding transcription factor 4 (Oct4), Forkhead Box A2 (FOXA2), FORSE-1, Brachyury, VE-cadherin, and α-smooth muscle actin (α-SMA)) of total protein were loaded on a 10% SDS-PAGE gel, transferred onto a polyvinyl difluoride membrane (PVDF, Merck Millipore; Darmstadt, Germany) and blocked in 5% low-fat milk in TBS-T buffer (Tris, 0.05% Tween20). The detection of selected proteins was then performed using the following specific primary and secondary antibodies: rabbit anti-HIF-1α (GeneTex, Irvine, CA, USA; GTX127309), mouse anti-MF20 (MHCα/β; antibody detects both alpha and beta MHC isoforms; kindly provided by Dr. Donald Fischman, Developmental Studies Hybridoma Bank, Iowa City, IA, USA), mouse anti-cTnT and rabbit anti-Brachyury (Abcam, Cambridge UK; ab33589; ab20680), rabbit anti-Oct4 and goat anti-FOXA2 (Santa Cruz, Santa Cruz, CA, USA; sc-9081; sc-6554), mouse anti-FORSE-1 (Hybridoma Bank), rabbit anti-VE-cadherin (Thermo Fisher Scientific; Waltham, MA USA; PA5-19612), mouse anti-α-SMA (Biomeda, Foster City, CA, USA; V1031), mouse anti-α-actinin (Sigma; St. Louis, MO, USA; A7811) and mouse anti-vinculin (Sigma; St. Louis, MO, USA; V9264) as a loading control. Bound antibodies were detected using anti-mouse and anti-rabbit HRP-conjugated secondary antibodies (both from Cell Signaling, Danvers, MA, USA; 7076S; 7074S). Optical densities were quantified by scanning densitometry and expressed in arbitrary units determined by ImageJ software (NIH, USA).

### Immunofluorescence staining

For immunofluorescent staining, 5+15d cells were digested with Accutase solution (Biowest; Nuaillé, France) for 5 min at 37°C and plated on Falcon Culture Slides (Corning Incorporated; New York, NY, USA) coated with 0.1% gelatin. After 24 hours of incubation, the cells were washed in 1x phosphate buffered saline (PBS), fixed in 0.5% formaldehyde for 20 minutes at room temperature (RT), permeabilized with 0.1% TritonX-100 for 30 min at RT, and then washed in 1xPBS. After 1 hour of blocking with 10% goat serum, the cells were incubated for 2h at RT with the following primary antibodies: mouse anti-MF20 (MHCα/β; antibody detects both alpha and beta MHC isoforms; kindly provided by Dr. Donald Fischman, Developmental Studies Hybridoma Bank, Iowa City, IA, USA) and mouse anti-cTnT (Abcam; Cambridge, UK; ab33589). Several washing steps later, the cells were incubated for 1h at RT with DyLight 488 conjugate goat anti-mouse IgG (Thermo Fisher Scientific; Waltham, MA USA; TF35502). Nuclei were counterstained with DAPI (1 μg/ml) and cells were mounted on microscopic slides in Mowiol (Calbiochem; La Jolla, CA, USA) solution (10% Mowiol 4–88 prepared in 25% glycerol, 100 mM Tris–HCl, and 0.6% 1.4-diazabicyclo-[2.2.2]-octane, pH 8.5). Images were acquired using a confocal microscope (TCS SP5; Leica; Wetzlar, Germany) equipped with a 63x1.4 oil immersion objective. The percentage of MHCα/β-positive cells were analyzed using a confocal microscope (Fluoview FV10i; Olympus; Shinjuku, Tokyo, Japan) and determined using ImageJ software (NIH, USA).

### Analysis of cells positive for cardiac markers

The percentage of mESC-derived cardiomyocytes (mESC-CMs) was quantified using flow cytometric analysis. 5+10d cells were digested using Accutase solution (specification above) and 1x10^6^ cells/ml were fixed and permeabilized using Cytofix/Cytoperm Fixation/Permeabilization Solution Kit (BD Biosciences; San Diego, CA, USA). The cells were then stained for 30 minutes with mouse anti-cTnT, rabbit anti-myosin light chain 2 (Myl2), or rabbit anti-myosin light chain 7 (Myl7) antibodies (all purchased from Abcam; Cambridge, UK; ab10214; ab92721; ab127001) and then incubated for 30 minutes with the appropriate secondary antibody: DyLight 488-conjugated goat anti-mouse IgG or DyLight 488 goat-anti rabbit IgG (Thermo Fisher Scientific; Waltham, MA USA; TF35502; TF35552). A minimum of 10,000 fixed cells were gated with the exclusion of non-single cells. The percentage of cTnT^+^, Myl7^+^, and Myl2^+^ cells in HIF-1α^+/+^ and HIF-1α^-/-^ were calculated. Analyses were performed using a FACSVerse flowcytometer (BD Biosciences; San Diego, CA, USA) and data were analyzed using Flowing Software 2. Results are shown as the fold change of HIF-1α^+/+^ cells.

### Transmission electron microscopy

Five-day-old EBs were seeded on a glass slide for the next 15 days (5+15d), fixed in 300 mM glutaraldehyde in 100 mM cacodylate buffer with 0.2% tannic acid, and post-fixed in 40 mM osmium tetroxide in 100 mM cacodylate buffer. Samples were dehydrated by increasing the concentration of ethanol (from 50 to 100%) and embedded in LR White embedding medium (Polysciences, Inc.; Warrington, PA, USA). Polymerization was carried out at 4°C using UV light. Ultrathin sections (60 nm thick) were cut using a Leica EM UC6 ultramicrotome and stained with uranyl acetate and lead citrate. Sections were examined under an FEI Morgagni 268D transmission electron microscope (FEI Company; Eindhoven, The Netherlands) [18].

### Analysis of cardiac cell beating

The beating characteristics of differentiated EBs were determined using CBAnalyser (for more details, see [19]). Briefly, the digital video of beating EBs or individual cells was recorded in phase contrast for approximately 5 minutes per sample using a digital camera (Olympus C-5060) mounted on an inverted microscope (Olympus IX41) at room temperature. The beating parameters were then analyzed from the recordings by CBAnalyser. Representative videos documenting the beating of HIF1alpha^+/+^ and HIF1alpha^-/-^ mESC-CMs (S1 Mov and S2 Mov), together with figures showing the analysis performed by CBAnalyser (S2 Fig) are included in the Supporting information files.

### Statistical analysis

Data are presented as mean ± standard error of the mean (SEM). Statistical differences between mean values were tested by one-way ANOVA with the Tukey HSD post hoc test and by Student’s t-test. P ≤ 0.05 was considered statistically significant.

## Results

### HIF-1α deficiency negatively affects cardiac differentiation

**Fig 2:**
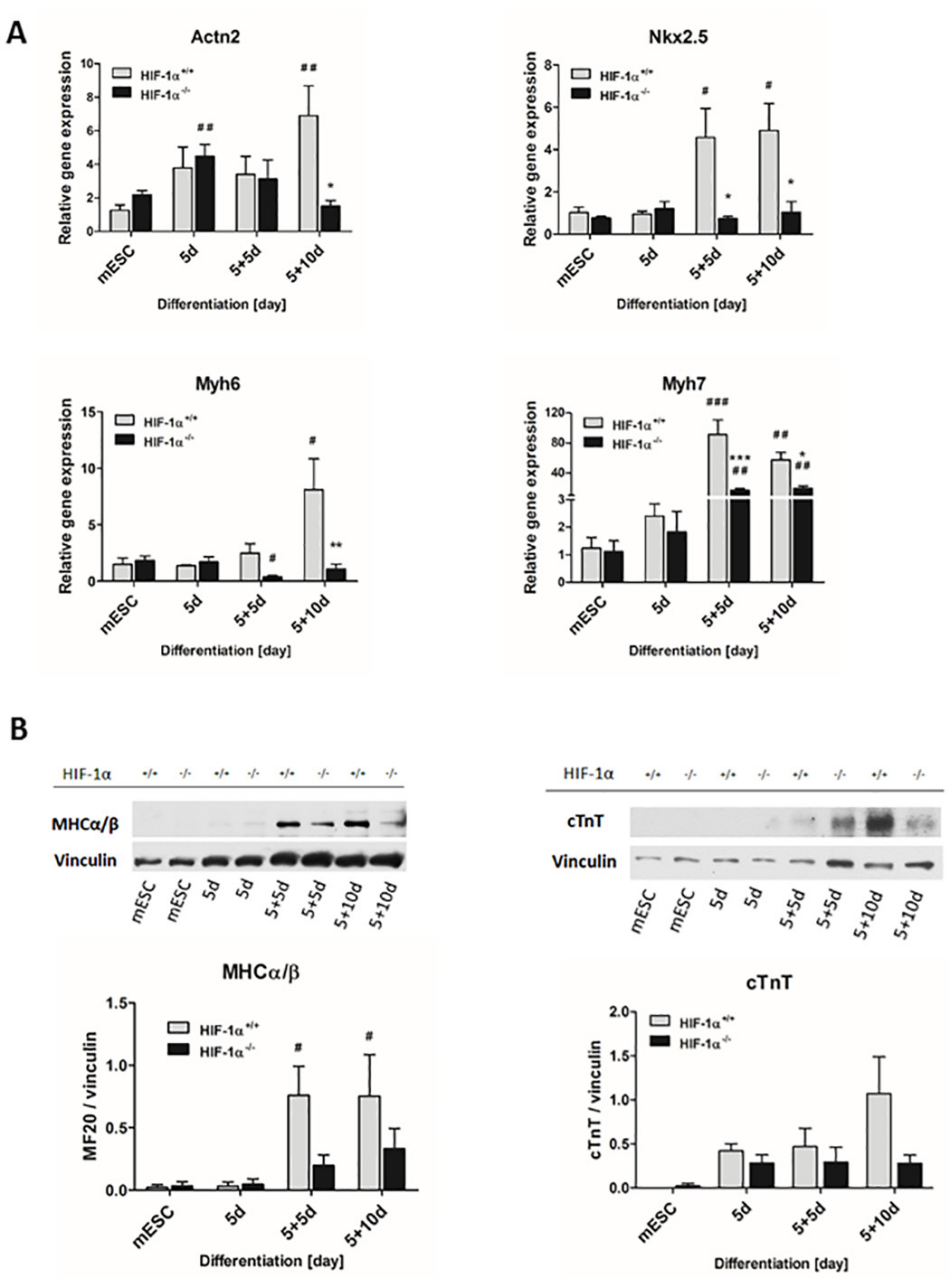
Effect of HIF-1α deficiency on cardiomyogenesis *in vitro*. (A) Relative gene expression of selected cardiac markers detected at mRNA level during cardiomyogenesis of wild type and HIF-1α-deficient mESC as revealed by real-time quantitative PCR analysis. The mRNA levels of α-actinin (*Actn2*), NK2 transcription factor related locus 5 (*Nkx2*.*5*), myosin heavy chain 6 (*Myh6*), and myosin heavy chain 7 (*Myh7*) were analyzed in undifferentiated ESC (mESC) and in 5-day-old EBs (5d) further differentiated for 5 or 10 days (5+5d, 5+10d). Data are presented as means ± SEM from at least 5 independent experiments; * p < 0.05, ** p < 0.01, *** p < 0.001 (HIF-1α^+/+^ vs. HIF-1α^-/-^ cells); # p < 0.05, ## p < 0.01, ### p < 0.001 (compared to parental mESC). (B) Protein levels of myosin heavy chains α and β (MHCα/β detected using anti-MF20 antibody) and cardiac troponin T were detected by western blot in the cells derived from wild type and HIF-1α deficient mESC and normalized to the vinculin signal. The densitometric analysis was performed on the basic of 4 independent experiments and data are presented as means ± SEM, # p < 0.05 (compared to parental mESC).

To evaluate the role of HIF-1α in the process of cardiomyogenesis, the model of HIF-1α^+/+^ and HIF-1α^-/-^ mESC differentiated via EB formation was employed. The progress of cardiomyogenic differentiation was characterized on the basis of changes in the expression of cardiac cell specific markers, such as NK2 transcription factor related locus 5 (*Nkx2*.*5*) [20], myosin heavy chain 6 and 7 (*Myh6*, *Myh7*)/MHCα/β [21], and Actn2 and cTnT [22]. The mRNA and protein levels of these markers were analyzed in undifferentiated mESC and in mESC differentiated for selected time intervals (5, 5+5d, 5+10d) (Fig 2).

The expression of all evaluated cardiac specific markers gradually increased during the differentiation of wild type HIF-1α^+/+^ mESC at both mRNA and protein levels, reaching their highest significant values generally in 5+5d (*Nkx2*.*5*, *Myh7*, MHCα/β) and 5+10d cells (*Actn2*, *Nkx2*.*5*, *Myh6*, *Myh7*, MHCα/β) (Fig 2A and 2B; gray bars). In contrast, equivalent analysis of HIF-1α^-/-^ cells did not reveal any significant enhancement of *Nkx2*.*5* or *Myh6* expression measured at mRNA levels during differentiation (Fig 2A; black bars). Interestingly, we observed a significant increase in *Actn2* expression in 5d-old HIF1α^-/-^ EBs; however, this pattern diminished with the progression of differentiation and eventually resulted in significant differences in *Actn2* mRNA levels between HIF1α^+/+^ and HIF1α^-/-^ 5+10d cells (Fig 2A). Finally, although we detected a significant increase in *Myh7* mRNA expression levels in HIF1α^-/-^ 5+5d and 5+10d cells (Fig 2A; black bars), their HIF1α^+/+^ counterparts displayed significantly higher levels of this cardiac specific marker (Fig 2A; gray bars). Mutual comparisons of the expression profiles of cardiac specific genes between HIF-1α^+/+^ and HIF-1α^-/-^ cells revealed similar trends only during the early stage of differentiation (5d) (Fig 2A). In contrast, in the late stages of cardiac cell development in 5+5d (*Nkx2*.*5 and Myh7*) and in 5+10d cells (all tested genes), the mRNA levels of these markers were significantly lower in HIF-1α^-/-^ cells (Fig 2A) and partially corresponded to western blot data showing lower protein levels of MHCα/β and cTnT (Fig 2B) as well as α-actinin (S3 Fig), when compared to their levels detected in HIF1α^+/+^ cells.

### HIF-1α deficiency is associated with lower cardiomyogenic maturation status

In order to characterize the maturation status of mESC-CMs derived from HIF-1α^+/+^ and HIF-1α^-/-^ cells, the intracellular localization of cTnT and myosin heavy chains (MHCα/β) was analyzed together with the ultrastructural features of the contractile apparatus. Further, cTnT and MHCα/β positive mESC-CMs were determined. Finally, the prevalence of atrial- or ventricular-like phenotypes of mESC-CMs was analyzed according to the expression profiles of atrial-specific *Myl7* and ventricular-specific *Myl2* genes [23].

**Fig 3:**
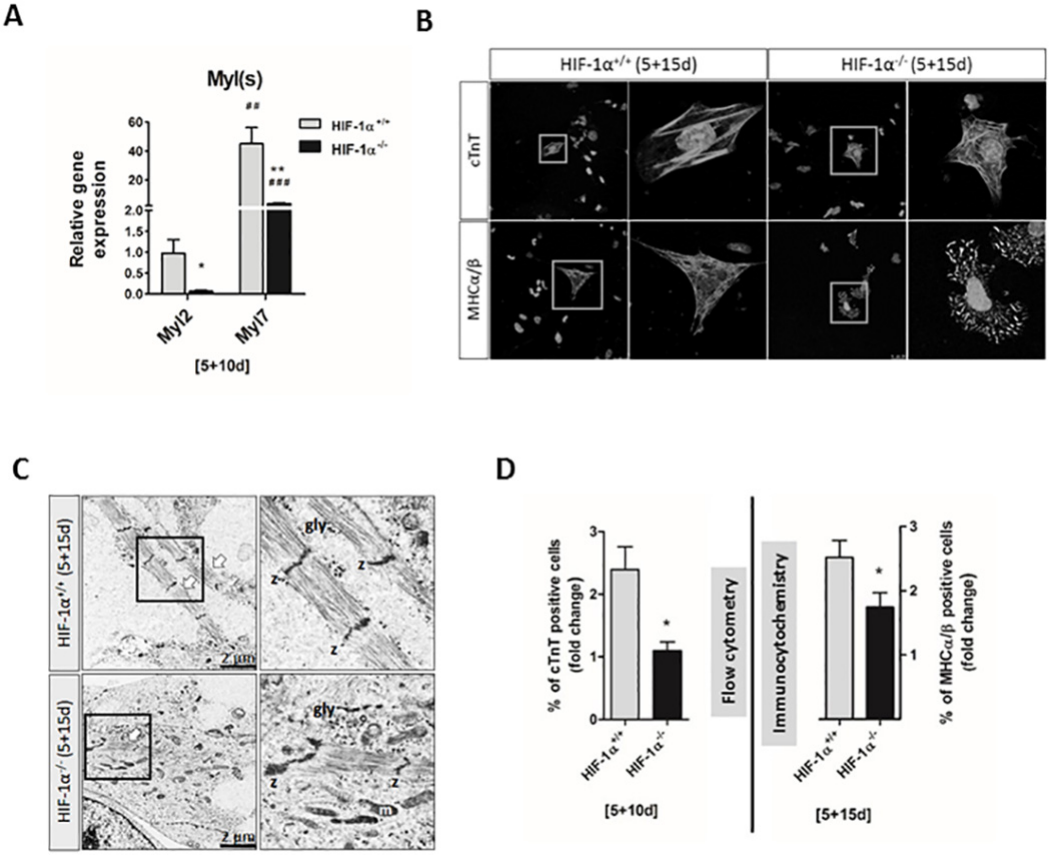
Characterization of wild type and HIF-1α deficient mESC-CMs. (A) The mRNA levels of myosin light chain 2 and 7 (*Myl2*, *Myl7*) genes as revealed by real-time quantitative PCR analysis. Data are presented as means ± SEM from at least 4 independent experiments; * p < 0.05, ** p < 0.01 (HIF-1α^+/+^ vs. HIF-1α^-/-^ cells); ## p < 0.01, ### p < 0.001 (compared to parental mESC). (B) The intracellular localization of cardiac troponin T (cTnT) and myosin heavy chain α and β (MHCα/β-using anti-MF20 antibody) proteins was specified by confocal microscopy in wild type and HIF-1α deficient differentiated cells (5+15d) processed from at least 3 independent experiments. (C) Transmission electron microscopy of wild type and HIF-1α deficient mESC-CMs at 5+15 day. Data are presented as means ± SEM from 4 independent experiments. The *arrows* indicate the presence of myofibrils, *z* Z-disk, *gly* glycogen and *m* mitochondria. (D) Analysis of the percentage of cardiac troponin T- positive cells (5+10d) performed by flow cytometry, and of myosin heavy chain α and β- (MHCα/β-using anti-MF20 antibody) positive cells (5+15d) performed by immunocytochemistry. Data are presented as means ± SEM of HIF-1α^+/+^ cell fold change from at least 3 independent experiments; * p < 0.05 (HIF-1α^+/+^ vs. HIF-1α^-/-^ cells).

Mutual comparison of *Myl7* and *Myl2* mRNA levels showed *Myl7* expression to be several orders higher compared to *Myl2* in both 5+10d HIF-1α^+/+^ and HIF-1α^-/-^ mESC-CMs (Fig 3A). Furthermore, the mRNA levels of *Myl2* and *Myl7* genes were significantly higher in HIF-1α^+/+^ mESC-CMs compared to HIF-1α^-/-^ mESC-CMs (Fig 3A). These findings correspond to the flow cytometric analysis showing a higher ratio of cells positive for ventricular-specific Myl2 to cells positive for atrial-specific Myl7 (0.45 ± 0.03) in HIF-1α^+/+^ mESC-CMs compared to HIF-1α^-/-^ mESC-CMs (0.29 ± 0.02) (S4 Fig).

Next, the intracellular localization of cTnT and MHCα/β was specified in 5+15d HIF-1α^+/+^ and HIF-1α^-/-^ mESC-CMs (Fig 3B). Although immunofluorescent staining showed similar localization patterns for cTnT in both HIF-1α^+/+^ and HIF-1α^-/-^ mESC-CMs, the uniform and fiber-like intracellular distribution of MHCα/β in HIF-1α^+/+^ mESC-CMs differed from the spot arrangement of these proteins in HIF-1α^-/-^ mESC-CMs.

Further, data from electron transmission microscopy revealed that both HIF-1α^+/+^ and HIF-1α^-/-^ mESC-CMs display ultrastructural features of early and non-fully mature cardiac cells (Fig 3C). However, in comparison with HIF-1α^-/-^ mESC-CMs differentiated for 20 days (5+15d), HIF-1α^+/+^ mESC-CMs revealed a greater degree of myofibril organization. The myofibrils exhibited straight segments formed by up to eight sarcomeres in HIF-1α^+/+^ mESC-CMs, and up to four in HIF-1α^-/-^ mESC-CMs. No other muscle-specific bands were visible in the sarcomere constructs, either in HIF-1α^+/+^ or HIF-1α^-/-^ mESC-CMs.

To further characterize the progress of cardiomyogenic differentiation, the percentage of mESC-CMs positive for cardiac specific markers cTnT and MHCα/β was analyzed on the basis of flowcytometric measurements (5+10d; cTnT) and immunocytochemistry (5+15d; MHCα/β) (Fig 3D). Data further support the importance of HIF1α, since there was a lower degree of cell positivity for cTnT and MHCα/β in HIF-1α^-/-^ mESC-CMs compared to HIF-1α^+/+^ mESC-CMs (Fig 3D).

### HIF-1α deficiency negatively affects the generation of beating embryoid bodies and cardiac cells

**Fig 4:**
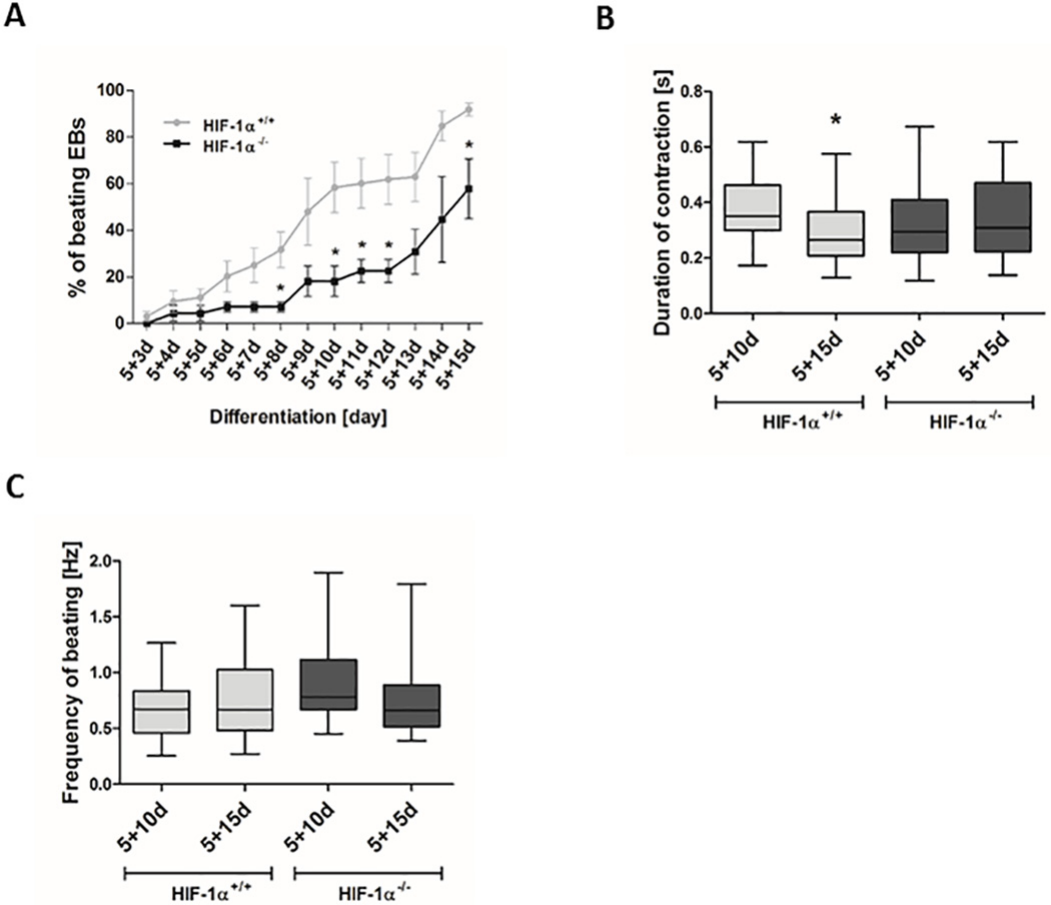
Effect of HIF-1α deficiency on the generation of beating embryonic bodies and cardiac cells. (A) Beating areas within EBs were counted from day 8 of differentiation (5+3d), (B) the duration of contraction and (C) frequency of beating was analyzed in both 5+10d, 5+15d cells and determined using CBAnalyser. Data are presented as means ± SEM from 3 independent experiments.

In order to further characterize the differences in cardiomyogenesis between HIF-1α^+/+^ and HIF-1α^-/-^ mESC, we determined the relative number of beating EBs, the duration of contraction, and the frequency of beating during cardiac differentiation (Fig 4A–4C). As shown in Fig 4A, the percentage of beating EBs decreased in HIF-1α^-/-^ mESC-CMs compared to HIF-1α^+/+^ mESC-CMs. Moreover, the advanced differentiation status of mESC-CMs was associated with a significantly shortened duration of contraction only in HIF-1α^+/+^ mESC-CMs (5+15d) compared to HIF-1α^+/+^ mESC-CMs differentiated for 5+10d (Fig 4B). However, we observed no differences in the frequency of beating between cells differentiated for 5+10d and 5+15d in both types of mESC-CMs (Fig 4C). These data describing the functional parameters of cardiomyocyte-like cells correspond with the observation that the cell positivity for the tested cardiac specific markers (cTnT, and MHCα/β) was lower in HIF-1α^-/-^ mESC-CMs compared to HIF-1α^+/+^ mESC-CMs (Fig 3D).

### HIFs are naturally stabilized in developing EBs during *in vitro* differentiation of mESC

**Fig 5:**
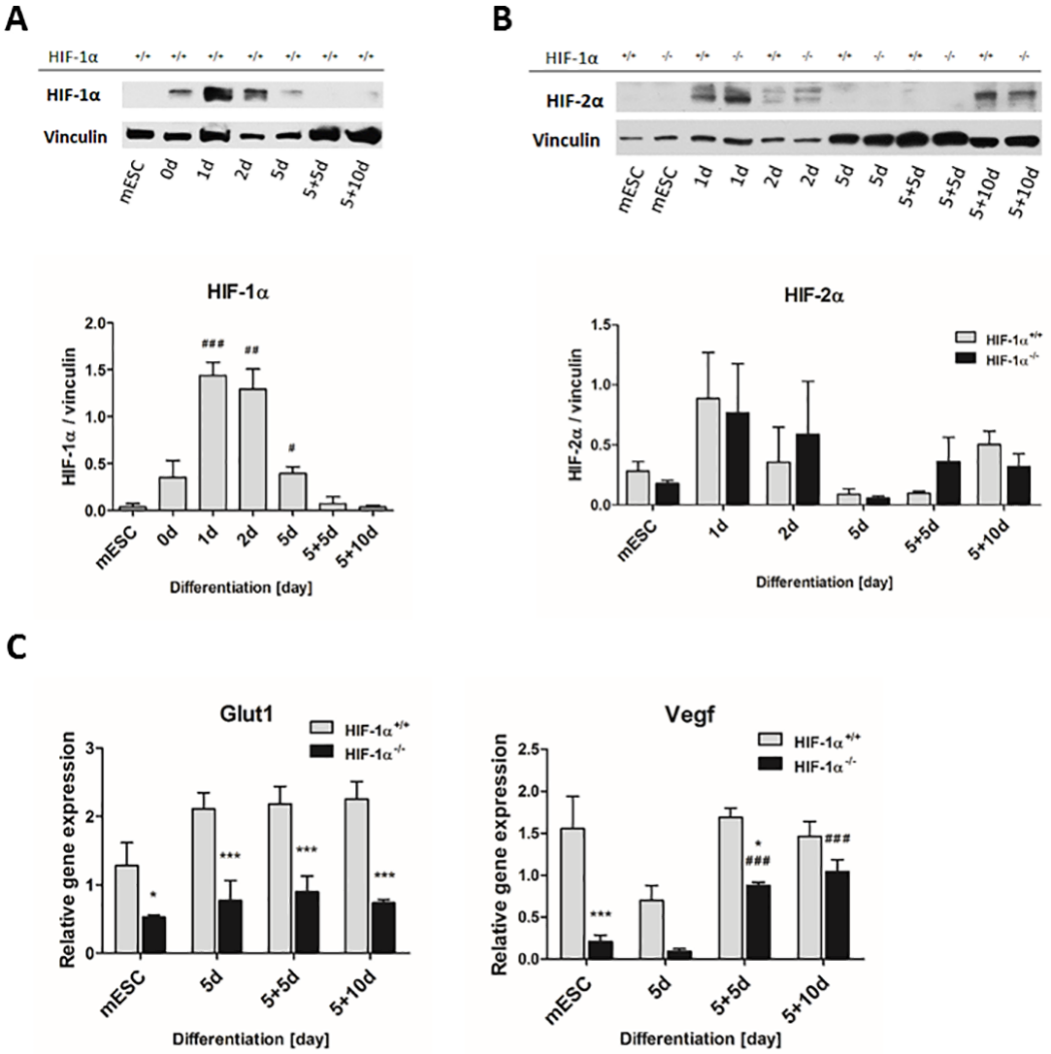
Stabilization and transcriptional activity of HIFs during *in vitro* differentiation. The protein levels of (A) HIF-1α and (B) HIF-2α were evaluated in wild type and HIF-1α deficient cells by western blot and normalized to the vinculin signal. Data are presented as means ± SEM. The densitometric analysis is representative of 3 independent experiments; # p < 0.05, ## p < 0.01, ### p < 0.001 (compared to parental mESC). (C) The mRNA levels of HIF-1α target genes glucose transporter 1 (*Glut1*) and vascular endothelial growth factor (*Vegf*) were measured by real-time quantitative PCR analysis. Data are presented as mean ± SEM from at least 4 independent experiments; * p < 0.05, *** p < 0.001 (HIF-1α^+/+^ vs. HIF-1α^-/-^ cells); ### p < 0.001 (compared to parental mESC).

The involvement of HIF-1α protein in the employed model of EB-mediated differentiation was proven by the protein stabilization of both HIF-1α and HIF-2α and by the expression profile of putative HIF-1α target genes (vascular endothelial growth factor (*Vegf*), and glucose transporter 1 (*Glut1*)) (Fig 5A–5C). Interestingly, the presence of HIF-1α protein had already appeared 24 hours after EB formation (0d) (Fig 5A). Further, the maximum level of HIF-1α protein stabilization was observed on day one (1d). From the second day of differentiation (2d), this stabilization rapidly decreased and reached a minimum in 10-day-old cells (5+5d). The lack of HIF-1α protein in HIF-1α^-/-^ EBs demonstrated the efficiency of HIF-1α gene deletion in these cells (S1 Fig). Considering the fact that HIF-1α and HIF-2α proteins may partially substitute each other in their functions [5], we also analyzed HIF-2α protein stabilization (Fig 5B). Similarly to HIF-1α, the HIF-2α protein signal reached a maximum on day 1 and then rapidly decreased. Interestingly, in the late stages of differentiation (5+5d and 5+10d), the level of HIF-2α protein stabilization again increased. Finally, statistical analysis of densitometric data did not reveal any significant differences between HIF-1α^+/+^ and HIF-1α^-/-^ cells at any time points (Fig 5B).

The expression profile of the *Glut1* gene was significantly enhanced in HIF-1α^+/+^ EBs compared to their HIF-1α^-/-^counterparts (Fig 5C). Similarly, at all observed time points, HIF-1α^+/+^ cells revealed a generally higher expression of *Vegf* in comparison with HIF-1α^-/-^ cells. Interestingly, *Vegf* expression significantly increased in HIF-1α^-/-^ EBs differentiated for 10 and 15 days.

### HIF1α deficiency reduces endoderm formation and attenuates the mESC differentiation of the cells of mesodermal origin

**Fig 6:**
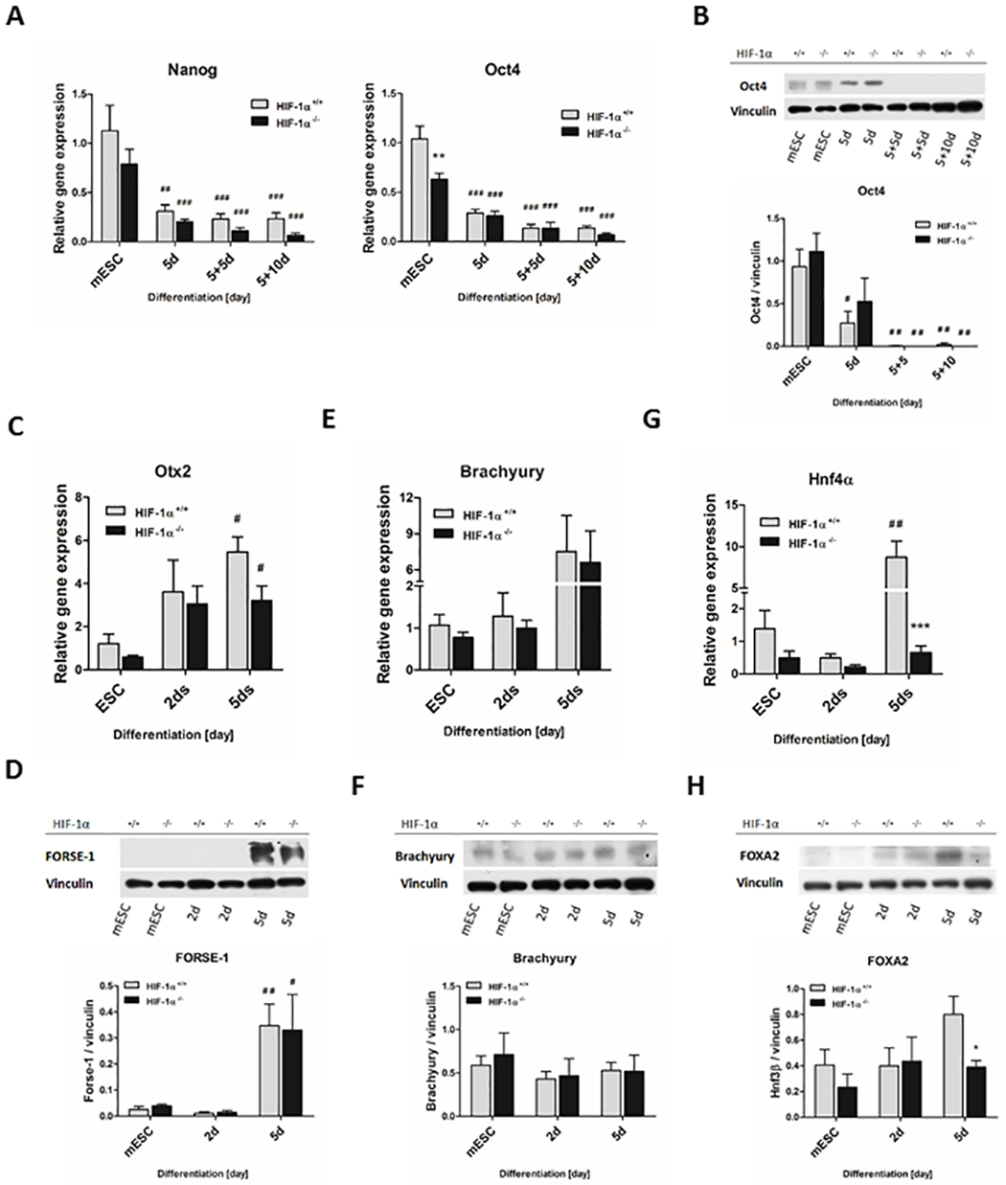
Effect of HIF-1α deficiency on lineage specification of spontaneously differentiating mESC. The mRNA levels of (A) embryonic stem cell markers *Nanog* and octamer-binding transcription factor 4 (*Oct4*); (C) ectoderm marker orthodenticle homolog 2 (*Otx2*); (E) mesoderm marker *Brachyury;* and (G) endoderm marker hepatic nuclear factor 4 α (*HNF4α*) were evaluated in wild type and HIF-1α deficient cells during the early stage of differentiation (mESC, 2d, 5d) using real-time quantitative PCR analysis. Data are presented as means ± SEM from at least 3 independent experiments; *** p < 0.001 (HIF-1α^+/+^ vs. HIF-1α^-/-^ cells); # p < 0.05, ## p < 0.01 (compared to parental mESC). The protein levels of (B) Oct4, (D) FORSE-1, (F) Brachyury and (H) Forkhead Box A2 (FOXA2) were determined by western blot analysis in the cells derived from wild type and HIF-1α deficient mESC and normalized to the vinculin signal. The densitometric analysis was performed on the basic of 3 independent experiments and data are presented as means ± SEM, * p < 0.05 (HIF-1α^+/+^ vs. HIF-1α^-/-^ cells); # p < 0.05, ## p < 0.01 (compared to parental mESC).

In order to verify the differentiation status of HIF-1α^+/+^ and HIF-1α^-/-^ cells, the expression profiles of the pluripotency markers *Oct4* and homeobox protein *Nanog* were analyzed (Fig 6A). The mRNA levels of both markers significantly decreased during the differentiation of both tested cell lines. HIF-1α^+/+^ mESC were characterized by the significantly higher gene expression of the *Oct4*. However, the increased gene expression of *Oct4* was not confirmed at the protein level (Fig 6B).

Regarding the negative effect of HIF-1α deficiency on cardiomyogenesis described above, we decided to analyze markers specific to mESC differentiation into all three germ layers at the specific time points (mESC, 2d, and 5d). The markers for endoderm (hepatic nuclear factor 4 α [*HNF4α;* FOXA2]), ectoderm (orthodenticle homolog 2 [*Otx2*]; FORSE-1), and mesoderm (*Brachyury*) were detected (Fig 6C–6H). The mRNA levels of *Otx2* and *Brachyury* gradually increased with the progression of differentiation in both HIF-1α^+/+^ and HIF-1α^-/-^ cells (Fig 6C and 6E). Moreover, the expression of *Otx2* was significantly upregulated on Day 5, similarly to the protein level of FORSE-1 (Fig 6D). However, the protein level of Brachyury did not change during differentiation in either cell line (Fig 6F). On the other hand, the mRNA levels of *Hnf4α* was increased (5d) after a slight decline on Day 2 (Fig 6G). Interestingly, mutual comparison of wild type and knockout cells showed a significant difference in the expression of *HNF4α* on Day 5. This was further supported by demonstration of the protein levels of another endodermal marker FOXA2 (Fig 6H).

**Fig 7:**
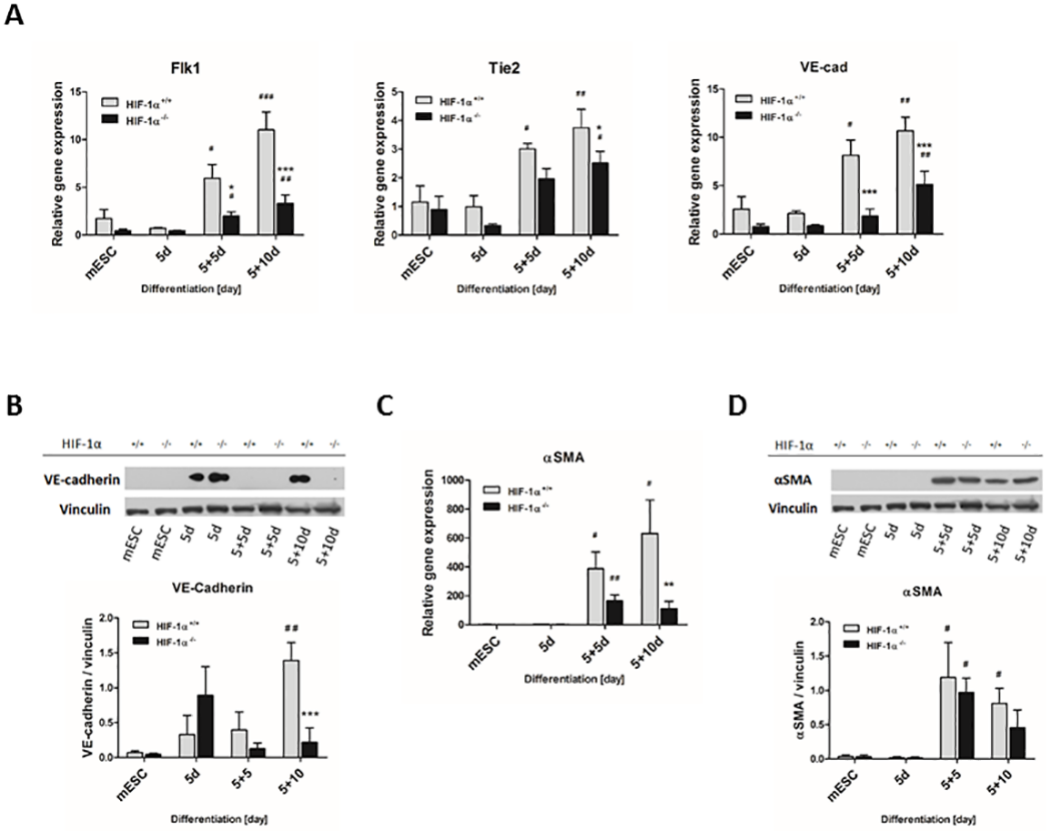
Effect of HIF-1α deficiency on cellular fate of spontaneously differentiating mESC. The gene expression profiles of (A) endothelial differentiation markers and (C) smooth muscle marker were analyzed during the differentiation process of wild type and HIF-1α deficient cells. The mRNA levels of (A) TEK tyrosine kinase (*Tie2*), VE-cadherin (*VE-cad*) and Fetal Liver Kinase 1 (*Flk1*) and (C) alpha smooth muscle actin (*αSMA*) were measured by real-time quantitative PCR analysis. Data are presented as means ± SEM from at least 5 independent experiments; * p < 0.05, ** p < 0.01, *** p < 0.001 (HIF-1α^+/+^ vs. HIF-1α^-/-^ cells); # p < 0.05, ## p < 0.01, ### p < 0.001 (compared to parental mESC). The protein levels of (B) VE-cadherin and (D) αSMA were determined by western blot analysis in the cells derived from wild type and HIF-1α deficient mESC and normalized to the vinculin signal. The densitometric analysis was performed on the basic of 3 independent experiments and data are presented as means ± SEM, *** p < 0.001 (HIF-1α^+/+^ vs. HIF-1α^-/-^ cells); # p < 0.05, ## p < 0.01 (compared to parental mESC).

To specify the effects of all observed findings, including the repression of endodermal as well as cardiac differentiation, the markers of other cell types in both HIF-1α^+/+^ and HIF-1α^-/-^ cells were analyzed (Fig 7A–7D), namely the markers for smooth muscle cells (*αSMA*), endothelial cells (TEK tyrosine kinase [*Tie2*], VE-cadherin [*VE-cad*], and fetal liver kinase 1 [*Flk1*]), and hematopoietic cells (*Gata1*, *PU*.*1*; data not shown). The mRNA levels of cell specific markers gradually increased along with the progression of HIF-1α^+/+^ cell differentiation, becoming significant already in 5+5d cells (*Tie2*, *Flk1*, *VE-cad*, *αSMA*) (Fig 7A and 7C). Similarly, 5+5d (*Flk1*, *αSMA*) and/or 5+10d (*Tie2*, *Flk1*, *αSMA*, *VE-cad*) HIF-1α^-/-^ cells also exhibited significant increases in the mRNA levels of the tested gene markers. However, mutual comparison of wild type and knockout cells revealed significantly lower expression profiles in HIF-1α^-/-^ 5+5d (*Flk1* and *VE-cad*) and in 5+10d (*Tie2*, *Flk1*, *VE-cad* and *αSMA*) cells. Further, the protein level of cell specific markers became significant on 5+5d (αSMA in both cell lines) and on 5+10d (VE-cadherin in HIF-1α^+/+^ mESC-CMs) (Fig 7B and 7D). Moreover, the level of VE-cadherin was significantly enhanced in wild type mESC-CMs on 5+10d compared to their knockout counterpart.

## Discussion

In this study, we demonstrated the importance of HIF-1α deletion for the spontaneous cardiac differentiation of mESC *in vitro*. Our data provide significant evidence that HIF-1α deletion negatively modulates both the quality and quantity of newly-formed and partially-differentiated mESC-CMs, as revealed by the attenuated expression profiles of cardiac specific markers in HIF-1α^-/-^ cells (*Nkx2*.*5*, *Actn2*, *Myh6*, *Myh7*, MHCα/β, cTnT) and by the lower percentage of HIF1α^-/-^ cells positive for specific cardiac markers (cTnT, MHCα/β). Furthermore, our data suggest the significant negative impact of HIF-1α deletion on the formation of endoderm, which participates in the signalization required for cardiomyocyte differentiation.

We carefully considered our own previously published protocols for mESC differentiation and also the protocols established by others [16, 19, 24] and employed an *in vitro* EB-based cellular model to investigate the effect of HIF-1α deficiency on spontaneous cardiomyogenesis (for a detailed scheme of the differentiation protocol, see Fig 1 and the Materials and methods section). Interestingly, we demonstrated that EB formation itself is associated with the natural stabilization of both HIF-1α and HIF-2α proteins and with the potentiation of HIF-related transcriptional activity (*Glut1*, *Vegf*). To our knowledge, this is the first time that the stabilization of both transcription factors in similar time frames has been demonstrated. Moreover, our data show similar profiles for HIF-2α protein stabilization during the differentiation of both HIF-1α^+/+^ and HIF-1α^-/-^ cells and, therefore, strongly suggest the particular lack of dependence of HIF-2α accumulation and its function on HIF-1α presence. Our results are consistent with the observations published by *Wartenberg et al*., who showed the transient up-regulation of HIF-1α in growing EBs up to the eighth day of differentiation [25]. In contrast, *Qi et al*. observed only HIF-2α stabilization [26].

It was previously shown, that the deletion of HIF-1α in ventricular cardiomyocytes led to several cardiac-specific developmental and morphological defects [2, 14]. On the other hand, the ectopic expression of HIF-1α protein was previously shown to promote the differentiation and maturation of cardiac cells derived from mESC (Ng, 2010). In accordance with these findings, our data show the deleterious effect of HIF-1α absence on spontaneous *in vitro* cardiomyogenesis. Further, we show for the first time that HIF-1α is associated with structural changes, which are reflected in the altered localization pattern of MHCα/β, essential components of the cardiomyocyte contractile apparatus, and in a reduction in the numbers and degree of organization of myofibrils. Moreover, we also show here for the first time that the presence of the HIF-1α protein is not essential for the occurrence of beating cardiomyocytes. Interestingly, the frequency of beating seems to be HIF-1α independent. However, in contrast to HIF-1α wild type cells, the duration of contraction in HIF-1α^-/-^ mESC-CMs does not accelerate during the late phase of differentiation. These results support our previous observation showing a decrease in the maturation status of HIF-1α^-/-^ mESC-CMs compared to their wild type counterpart. This is in complete agreement with the *in vivo* results [14]. However, it is partially in contrast with the *in vitro* study published by *Ateghang at al*. [27], who showed the complete absence of beating cardiomyocytes in 11-day-old EBs differentiated from HIF-1α^-/-^ mESC. This inconsistency could be partially explained by the differences in mESC differentiation protocols and thus by the discrepancy in the maturation status of the analyzed mESC-CMs. Nevertheless, previously published studies and our data enable us to suggest that HIF-1α is one of the key factors regulating the differentiation and maturation of mESC-CMs [12, 28].

Heart development is further characterized by the progressive metabolic shift of cardiac cells from fetal to adult gene programming [29]. While the fetal phenotype is characterized by high regenerative capacity, anaerobic glycolysis, and the predominant expression of *Myh7*, the adult phenotype is associated with extremely limited cardiomyocyte renewal, oxidative phosphorylation, and the predominant expression of *Myh6* [30, 31]. Therefore, our observations that mESC-CMs are specified by the predominant expression of *Myh7*, by the presence of non-fully maturated myofibrils (premyofibrils or nascent myofibrils) [32], and by the high potential of cells to differentiate preferentially into atrial-like cardiomyocytes are of particular importance. Furthermore, these phenomena are promoted by HIF-1α deficiency. Taking all these results into consideration, we can conclude that our data strongly indicate the non-fully maturated state of both wild type and HIF-1α-deficient mESC-CMs, which exhibit features similar to those of fetal cardiomyocytes. Moreover, besides the characterization of cardiomyocytes on the basis of their maturation and metabolic status, the expression profiles of markers suitable for distinguishing between atrial-like and ventricular-like cardiomyocytes were also evaluated, since this knowledge can provide us with further insights into the functional fate of mESC-derived cardiac-like cells [23]. We demonstrated a lower ratio of ventricular-specific Myl2 mESC-CMs to cells positive for atrial-specific Myl7 in HIF-1α^+/+^ compared to HIF-1α^-/-^ cells. This finding tempts us to speculate, that besides having a general impact on the formation and maturation of mES-CMs, HIF1α protein might also modulate the atrial/ventricular fate of cardiac-like cells and thus their functional properties [33].

Spontaneous cardiomyogenesis via EBs is associated not only with the formation of other cell types of mesodermal origin including fibroblast, endothelial, and smooth muscle cells, but also with the early formation of endoderm and ectoderm layers [34, 35]. Recent publications have provide clear evidence that specific endoderm signalization is required for cardiomyogenic cell fate [35–38]. The role of essential cardiomyogenic factor BMP2, which is specifically secreted by the visceral endoderm, has been documented in depth [35, 36, 39]. Importantly, our data clearly demonstrate that HIF-1α deletion has a negative effect on the endoderm formation, which is probably closely related with the decrease of cardiomyogenesis *in vitro*. However, the further study is required. Furthermore, based on the expression profiles of differentiated mESC, our study brings new important evidence, that HIF-1α-deficient cells do not prefer differentiation to other cell types of a mesoderm origin. Interestingly, both the development and maturation of cardiomyocytes was previously described to be essentially dependent on the establishment of intercellular endothelial-cardiomyocyte interactions [40]. The observation that HIF-1α deficiency negatively regulates also the gene expressions of endothelial cell markers (Flk1, Tie2, VE-cadherin) as well as the protein levels of VE-cadherin is therefore of particular interest and, together with our data presented above, point to the complex importance of HIF-1α for cardiac cell development.

**Fig 8:**
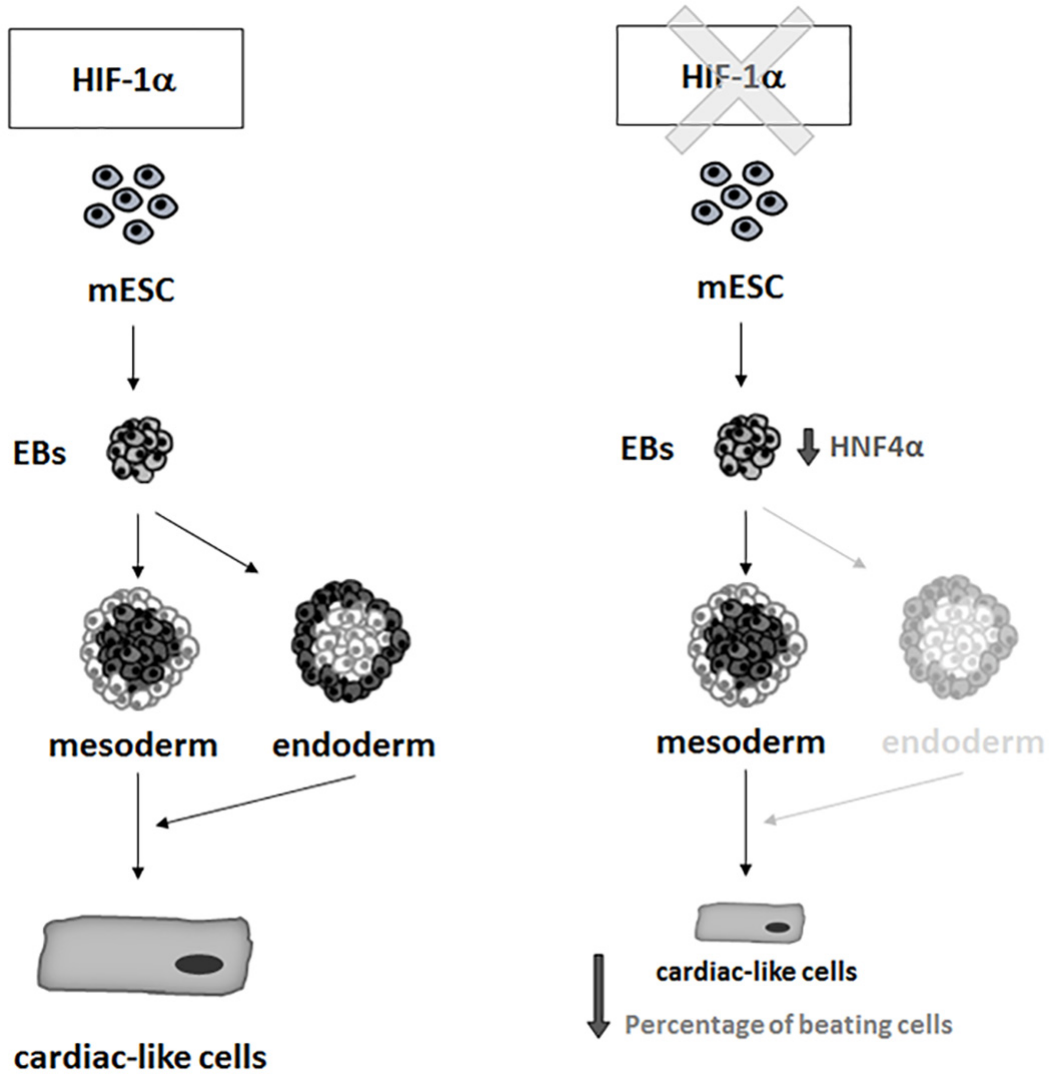
Scheme indicating the possible role of HIF-1α in the regulation of cardiomyogenesis *in vitro*. For more details see discussion.

In conclusion, this study presents the first evidence of the role of HIF-1α in the differentiation and maturation of mESC-derived CMs *in vitro* (Fig 8). HIF-1α deficiency negatively affects the differentiation and maturation of cardiac-like cells, as well as the percentage of these newly formed cells. Interestingly, this phenomenon is further associated with the reduced formation of endoderm. These results may provide a novel perspective on cardiac differentiation and offer a useful means of studying cardiac regeneration induced by various pathophysiological conditions associated, for example, with hypoxic stress.

## Supporting Information

S1 Fig The protein levels of HIF-1α. The representative western blot analysis of the protein levels of HIF-1α protein in wild type and HIF-1α deficient mESC during the early phase of differentiation. The protein levels of the vinculin is shown.
(PDF)

S2 Fig The representative figures showing the analysis performed by the CBAnalyser. (PDF)

S3 Fig The protein levels of α-actinin. The protein levels of α-actinin were evaluated in wild type and HIF-1α deficient cells by western blot and normalized to the vinculin signal. Data are presented as means ± SEM. The densitometric analysis is representative of 3 independent experiments.
(PDF)

S4 Fig The ratio of cells positive for ventricular-specific myosin light chain 2 (Myl2) to cells positive for atrial-specific myosin light chain 7 (Myl7). The ratio of cells positive for Myl2 to cells positive for Myl7 that was determined by flow cytometric analysis. Data are presented as means ± SEM from at least 3 independent experiments.
(PDF)

S1 Mov Representative video documenting the beating of HIF1alpha+/+ mESC-CMs. (MOV)

S2 Mov Representative video documenting the beating of HIF1alpha-/- mESC-CMs. (MOV)

## References

[1] Braunwald E, Bristow MR. Congestive heart failure: fifty years of progress. Circulation. 2000;102(20 Suppl 4):IV14–23. Epub 2000/11/18. . 11080127 10.1161/01.cir.102.suppl_4.iv-14

[2] Krishnan J, Ahuja P, Bodenmann S, Knapik D, Perriard E, Krek W, et al Essential role of developmentally activated hypoxia-inducible factor 1alpha for cardiac morphogenesis and function. Circ Res. 2008;103(10):1139–46. Epub 2008/10/14. 10.1161/01.RES.0000338613.89841.c1 . 18849322

[3] Medley TL, Furtado M, Lam NT, Idrizi R, Williams D, Verma PJ, et al Effect of oxygen on cardiac differentiation in mouse iPS cells: role of hypoxia inducible factor-1 and Wnt/beta-catenin signaling. PLoS One. 2013;8(11):e80280 Epub 2013/11/23. 10.1371/journal.pone.0080280 24265804 PMC3827186

[4] Majmundar AJ, Wong WJ, Simon MC. Hypoxia-inducible factors and the response to hypoxic stress. Mol Cell. 2010;40(2):294–309. Epub 2010/10/23. 10.1016/j.molcel.2010.09.022 20965423 PMC3143508

[5] Hu CJ, Wang LY, Chodosh LA, Keith B, Simon MC. Differential roles of hypoxia-inducible factor 1alpha (HIF-1alpha) and HIF-2alpha in hypoxic gene regulation. Mol Cell Biol. 2003;23(24):9361–74. Epub 2003/12/04. 14645546 10.1128/MCB.23.24.9361-9374.2003 PMC309606

[6] Wang GL, Jiang BH, Rue EA, Semenza GL. Hypoxia-inducible factor 1 is a basic-helix-loop-helix-PAS heterodimer regulated by cellular O2 tension. Proc Natl Acad Sci U S A. 1995;92(12):5510–4. Epub 1995/06/06. 7539918 10.1073/pnas.92.12.5510 PMC41725

[7] Gonzalez IR, Moreno-Manzano V, Rodriguez-Jimenez FJ, Sepulveda P, Sanchez-Puelles JM. The biology of HIFalpha proteins in cell differentiation and disease. Vitam Horm. 2011;87:367–79. Epub 2011/12/01. 10.1016/B978-0-12-386015-6.00036-6 . 22127251

[8] Ema M, Taya S, Yokotani N, Sogawa K, Matsuda Y, Fujii-Kuriyama Y. A novel bHLH-PAS factor with close sequence similarity to hypoxia-inducible factor 1alpha regulates the VEGF expression and is potentially involved in lung and vascular development. Proc Natl Acad Sci U S A. 1997;94(9):4273–8. Epub 1997/04/29. 9113979 10.1073/pnas.94.9.4273 PMC20712

[9] Wiesener MS, Jurgensen JS, Rosenberger C, Scholze CK, Horstrup JH, Warnecke C, et al Widespread hypoxia-inducible expression of HIF-2alpha in distinct cell populations of different organs. FASEB journal: official publication of the Federation of American Societies for Experimental Biology. 2003;17(2):271–3. Epub 2002/12/20. 10.1096/fj.02-0445fje . 12490539

[10] Iyer NV, Kotch LE, Agani F, Leung SW, Laughner E, Wenger RH, et al Cellular and developmental control of O2 homeostasis by hypoxia-inducible factor 1 alpha. Genes Dev. 1998;12(2):149–62. Epub 1998/03/07. 9436976 10.1101/gad.12.2.149 PMC316445

[11] Scortegagna M, Ding K, Oktay Y, Gaur A, Thurmond F, Yan LJ, et al Multiple organ pathology, metabolic abnormalities and impaired homeostasis of reactive oxygen species in Epas1-/- mice. Nat Genet. 2003;35(4):331–40. Epub 2003/11/11. 10.1038/ng1266 . 14608355

[12] Ng KM, Lee YK, Chan YC, Lai WH, Fung ML, Li RA, et al Exogenous expression of HIF-1 alpha promotes cardiac differentiation of embryonic stem cells. J Mol Cell Cardiol. 2010;48(6):1129–37. Epub 2010/02/02. 10.1016/j.yjmcc.2010.01.015 . 20116384

[13] Sun X, Pang L, Shi M, Huang J, Wang Y. HIF2alpha induces cardiomyogenesis via Wnt/beta-catenin signaling in mouse embryonic stem cells. J Transl Med. 2015;13:88 Epub 2015/04/19. 10.1186/s12967-015-0447-7 25889500 PMC4399227

[14] Huang Y, Hickey RP, Yeh JL, Liu D, Dadak A, Young LH, et al Cardiac myocyte-specific HIF-1alpha deletion alters vascularization, energy availability, calcium flux, and contractility in the normoxic heart. FASEB journal: official publication of the Federation of American Societies for Experimental Biology. 2004;18(10):1138–40. Epub 2004/05/11. 10.1096/fj.04-1510fje . 15132980

[15] Carmeliet P, Dor Y, Herbert JM, Fukumura D, Brusselmans K, Dewerchin M, et al Role of HIF-1alpha in hypoxia-mediated apoptosis, cell proliferation and tumour angiogenesis. Nature. 1998;394(6692):485–90. Epub 1998/08/11. 10.1038/28867 . 9697772

[16] Dahlmann J, Kensah G, Kempf H, Skvorc D, Gawol A, Elliott DA, et al The use of agarose microwells for scalable embryoid body formation and cardiac differentiation of human and murine pluripotent stem cells. Biomaterials. 2013;34(10):2463–71. Epub 2013/01/22. 10.1016/j.biomaterials.2012.12.024 . 23332176

[17] Livak KJ, Schmittgen TD. Analysis of relative gene expression data using real-time quantitative PCR and the 2(-Delta Delta C(T)) Method. Methods. 2001;25(4):402–8. Epub 2002/02/16. 10.1006/meth.2001.1262 . 11846609

[18] Lundy SD, Zhu WZ, Regnier M, Laflamme MA. Structural and functional maturation of cardiomyocytes derived from human pluripotent stem cells. Stem Cells Dev. 2013;22(14):1991–2002. Epub 2013/03/07. 10.1089/scd.2012.0490 23461462 PMC3699903

[19] Radaszkiewicz KA, Sykorova D, Karas P, Kudova J, Kohut L, Bino L, et al Simple non-invasive analysis of embryonic stem cell-derived cardiomyocytes beating in vitro. The Review of scientific instruments. 2016;87(2):024301 Epub 2016/03/05. 10.1063/1.4941776 . 26931869

[20] Sepulveda JL, Belaguli N, Nigam V, Chen CY, Nemer M, Schwartz RJ. GATA-4 and Nkx-2.5 coactivate Nkx-2 DNA binding targets: role for regulating early cardiac gene expression. Mol Cell Biol. 1998;18(6):3405–15. Epub 1998/06/20. 9584181 10.1128/mcb.18.6.3405 PMC108922

[21] Lyons GE, Schiaffino S, Sassoon D, Barton P, Buckingham M. Developmental regulation of myosin gene expression in mouse cardiac muscle. J Cell Biol. 1990;111(6 Pt 1):2427–36. Epub 1990/12/01. 2277065 10.1083/jcb.111.6.2427 PMC2116419

[22] Sjoblom B, Salmazo A, Djinovic-Carugo K. Alpha-actinin structure and regulation. Cell Mol Life Sci. 2008;65(17):2688–701. Epub 2008/05/20. 10.1007/s00018-008-8080-8 . 18488141 PMC11131806

[23] Kubalak SW, Miller-Hance WC, O'Brien TX, Dyson E, Chien KR. Chamber specification of atrial myosin light chain-2 expression precedes septation during murine cardiogenesis. J Biol Chem. 1994;269(24):16961–70. Epub 1994/06/17. . 8207020

[24] Pesl M, Acimovic I, Pribyl J, Hezova R, Vilotic A, Fauconnier J, et al Forced aggregation and defined factors allow highly uniform-sized embryoid bodies and functional cardiomyocytes from human embryonic and induced pluripotent stem cells. Heart and vessels. 2014;29(6):834–46. Epub 2013/11/22. 10.1007/s00380-013-0436-9 . 24258387

[25] Wartenberg M, Donmez F, Ling FC, Acker H, Hescheler J, Sauer H. Tumor-induced angiogenesis studied in confrontation cultures of multicellular tumor spheroids and embryoid bodies grown from pluripotent embryonic stem cells. FASEB journal: official publication of the Federation of American Societies for Experimental Biology. 2001;15(6):995–1005. Epub 2001/04/09. . 11292660 10.1096/fj.00-0350com

[26] Qi Y, Tian X, Liu J, Han Y, Graham AM, Simon MC, et al Bnip3 and AIF cooperate to induce apoptosis and cavitation during epithelial morphogenesis. J Cell Biol. 2012;198(1):103–14. Epub 2012/07/04. 10.1083/jcb.201111063 22753893 PMC3392936

[27] Ateghang B, Wartenberg M, Gassmann M, Sauer H. Regulation of cardiotrophin-1 expression in mouse embryonic stem cells by HIF-1alpha and intracellular reactive oxygen species. Journal of cell science. 2006;119(Pt 6):1043–52. Epub 2006/03/02. 10.1242/jcs.02798 . 16507596

[28] Bianco C, Cotten C, Lonardo E, Strizzi L, Baraty C, Mancino M, et al Cripto-1 is required for hypoxia to induce cardiac differentiation of mouse embryonic stem cells. Am J Pathol. 2009;175(5):2146–58. Epub 2009/10/17. 10.2353/ajpath.2009.090218 19834060 PMC2774077

[29] Fukushima A, Milner K, Gupta A, Lopaschuk GD. Myocardial Energy Substrate Metabolism in Heart Failure: from Pathways to Therapeutic Targets. Curr Pharm Des. 2015;21(25):3654–64. Epub 2015/07/15. . 26166604 10.2174/1381612821666150710150445

[30] Choi WY, Poss KD. Cardiac regeneration. Curr Top Dev Biol. 2012;100:319–44. Epub 2012/03/28. 10.1016/B978-0-12-387786-4.00010-5 22449849 PMC3342383

[31] Rajabi M, Kassiotis C, Razeghi P, Taegtmeyer H. Return to the fetal gene program protects the stressed heart: a strong hypothesis. Heart Fail Rev. 2007;12(3–4):331–43. Epub 2007/05/23. 10.1007/s10741-007-9034-1 . 17516164

[32] Dabiri GA, Turnacioglu KK, Sanger JM, Sanger JW. Myofibrillogenesis visualized in living embryonic cardiomyocytes. Proc Natl Acad Sci U S A. 1997;94(17):9493–8. Epub 1997/08/19. 9256510 10.1073/pnas.94.17.9493 PMC23235

[33] Maltsev VA, Rohwedel J, Hescheler J, Wobus AM. Embryonic stem cells differentiate in vitro into cardiomyocytes representing sinusnodal, atrial and ventricular cell types. Mech Dev. 1993;44(1):41–50. Epub 1993/11/01. . 8155574 10.1016/0925-4773(93)90015-p

[34] Banerjee I, Fuseler JW, Price RL, Borg TK, Baudino TA. Determination of cell types and numbers during cardiac development in the neonatal and adult rat and mouse. Am J Physiol Heart Circ Physiol. 2007;293(3):H1883–91. Epub 2007/07/03. 10.1152/ajpheart.00514.2007 . 17604329

[35] Van Vliet P, Wu SM, Zaffran S, Puceat M. Early cardiac development: a view from stem cells to embryos. Cardiovascular research. 2012;96(3):352–62. Epub 2012/08/16. 10.1093/cvr/cvs270 22893679 PMC3500045

[36] Madabhushi M, Lacy E. Anterior visceral endoderm directs ventral morphogenesis and placement of head and heart via BMP2 expression. Developmental cell. 2011;21(5):907–19. Epub 2011/11/15. 10.1016/j.devcel.2011.08.027 22075149 PMC3386144

[37] Liang S, Li HC, Wang YX, Wu SS, Cai YJ, Cui HL, et al Pulmonary endoderm, second heart field and the morphogenesis of distal outflow tract in mouse embryonic heart. Development, growth & differentiation. 2014;56(4):276–92. Epub 2014/04/05. 10.1111/dgd.12129 . 24697670

[38] Youngblood BA, MacDonald CC. CstF-64 is necessary for endoderm differentiation resulting in cardiomyocyte defects. Stem cell research. 2014;13(3 Pt A):413–21. Epub 2014/12/03. 10.1016/j.scr.2014.09.005 25460602 PMC4319989

[39] Lickert H, Kutsch S, Kanzler B, Tamai Y, Taketo MM, Kemler R. Formation of multiple hearts in mice following deletion of beta-catenin in the embryonic endoderm. Developmental cell. 2002;3(2):171–81. Epub 2002/08/27. . 12194849 10.1016/s1534-5807(02)00206-x

[40] Chen K, Bai H, Arzigian M, Gao YX, Bao J, Wu WS, et al Endothelial cells regulate cardiomyocyte development from embryonic stem cells. Journal of cellular biochemistry. 2010;111(1):29–39. Epub 2010/05/28. 10.1002/jcb.22680 20506197 PMC2930113

