# Supplementary material for: HIF-1alpha Deficiency Attenuates the Cardiomyogenesis of Mouse Embryonic Stem Cells
Source: PLoS One. 2016 Jun 29;11(6):e0158358. doi: 10.1371/journal.pone.0158358 (PMC4927095; doi:10.1371/journal.pone.0158358)

Supporting information

Figure S1: The representative western blot analysis of the HIF-1alpha protein stabilization in in wild type and HIF-1α deficient mESC during the early phase of differentiation.

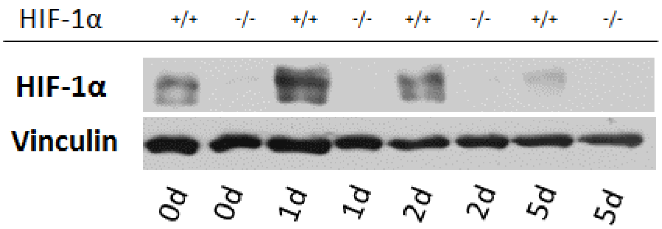

Supplement: S1 Fig — The representative western blot analysis of the protein levels of HIF-1α protein in wild type and HIF-1α deficient mESC during the early phase of differentiation. The protein levels of the vinculin is shown. (PDF) [file pone.0158358.s001.pdf]
