# Supplementary material for: HIF-1alpha Deficiency Attenuates the Cardiomyogenesis of Mouse Embryonic Stem Cells
Source: PLoS One. 2016 Jun 29;11(6):e0158358. doi: 10.1371/journal.pone.0158358 (PMC4927095; doi:10.1371/journal.pone.0158358)

Supporting information

Figure S2: The representative figures showing the analysis of 5+15d mESC-CMs beating performed by the CBAnalyser.

HIF-1α$^{+/+}$ HIF-1α$^{-/-}$

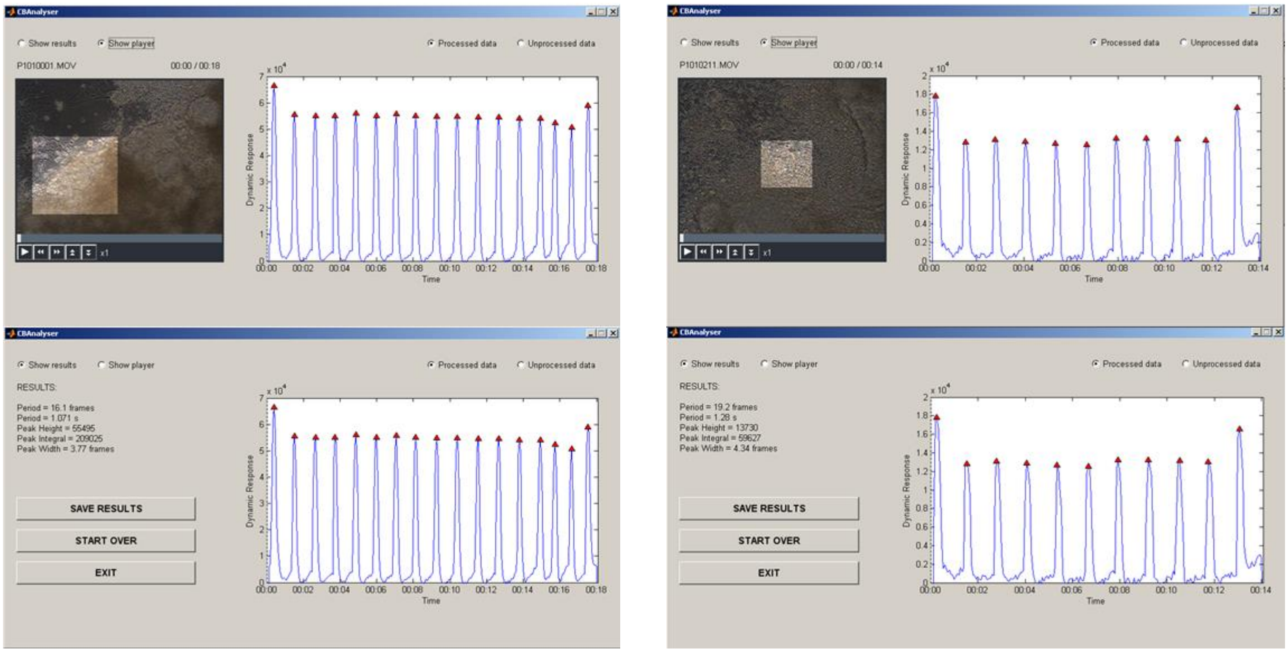

Supplement: S2 Fig — (PDF) [file pone.0158358.s002.pdf]
