# Supplementary material for: HIF-1alpha Deficiency Attenuates the Cardiomyogenesis of Mouse Embryonic Stem Cells
Source: PLoS One. 2016 Jun 29;11(6):e0158358. doi: 10.1371/journal.pone.0158358 (PMC4927095; doi:10.1371/journal.pone.0158358)
Supplement: S3 Fig — The protein levels of α-actinin were evaluated in wild type and HIF-1α deficient cells by western blot and normalized to the vinculin signal. Data are presented as means ± SEM. The densitometric analysis is representative of 3 independent experiments. (PDF) [file pone.0158358.s003.pdf]

Supporting information

Figure S3: The protein levels of alpha-actinin.

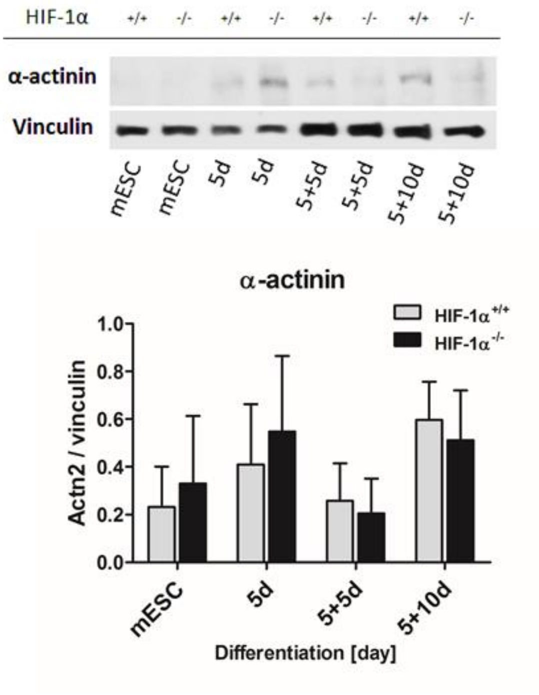

Data are presented as means ± SEM. The densitometric analysis is representative of 3 independent experiments.

Supporting information

Figure S4: The ratio of cells positive for ventricular-specific Myl2 to cells positive for atrial-specific Myl7

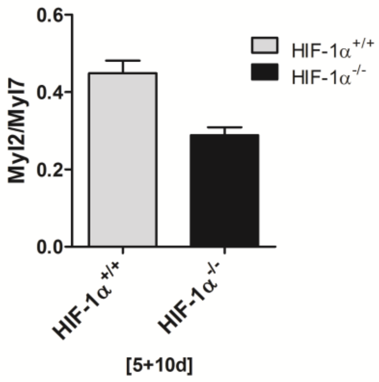

Flow cytometric analysis showing the ratio of cells positive for ventricular-specific myosin light chain 2 (Myl2) to cells positive for atrial-specific myosin light chain 7 (Myl7). Data are presented as means ± SEM from at least 3 independent experiments.

Supporting information

Movie S1: Video record of beating cardiomyocytes derived from HIF-1alpha wild type mESC

Supporting information

Movie S2: Video record of beating cardiomyocytes derived from HIF-1alpha knockout mESC
