# Supplementary material for: HIF-1alpha Deficiency Attenuates the Cardiomyogenesis of Mouse Embryonic Stem Cells
Source: PLoS One. 2016 Jun 29;11(6):e0158358. doi: 10.1371/journal.pone.0158358 (PMC4927095; doi:10.1371/journal.pone.0158358)
Supplement: S4 Fig — The ratio of cells positive for Myl2 to cells positive for Myl7 that was determined by flow cytometric analysis. Data are presented as means ± SEM from at least 3 independent experiments. (PDF) [file pone.0158358.s004.pdf]

Supporting information

Figure S4: The ratio of cells positive for ventricular-specific Myl2 to cells positive for atrial-specific Myl7

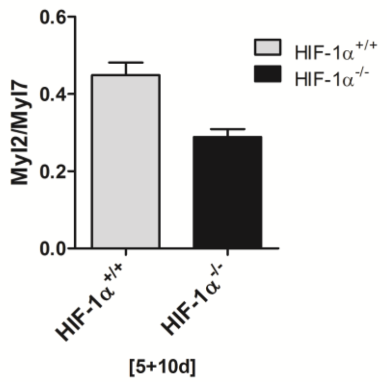

Flow cytometric analysis showing the ratio of cells positive for ventricular-specific myosin light chain 2 (Myl2) to cells positive for atrial-specific myosin light chain 7 (Myl7). Data are presented as means ± SEM from at least 3 independent experiments.
